# Doping of metal–organic frameworks towards resistive sensing

**DOI:** 10.1038/s41598-017-02618-y

**Published:** 2017-05-26

**Authors:** Hidetsugu Shiozawa, Bernhard C. Bayer, Herwig Peterlik, Jannik C. Meyer, Wolfgang Lang, Thomas Pichler

**Affiliations:** Faculty of Physics, University of Vienna, Boltzmanngasse 5, 1090 Vienna, Austria

## Abstract

Coordination polymerization leads to various metal–organic frameworks (MOFs) with unique physical properties and chemical functionalities. One of the challenges towards their applications as porous materials is to make MOFs optimally conductive to be used as electronic components. Here, it is demonstrated that Co-MOF-74, a honeycomb nano–framework with one–dimensionally arranged cobalt atoms, advances its physical properties by accommodating tetracyanochinodimethan (TCNQ), an acceptor molecule. Strong intermolecular charge transfer reduces the optical band gap down to 1.5 eV of divalent TCNQ and enhances the electrical conduction, which allows the MOF to be utilized for resistive gas- and photo-sensing. The results provide insight into the electronic interactions in doped MOFs and pave the way for their electronic applications.

## Introduction

Metal–organic frameworks (MOFs)^1, 2^ are exciting materials due to their unprecedented nanostructures that can be tailored on a bulk scale by linking designated metal ligands and organic molecules. Their colourful appearances that can change for instance with solvent exchange and molecular doping^3^ indicates their chromophoric nature. Also, the MOFs’ diverse physical and chemical tunability is promising as demonstrated via building block replacement^4^ and infiltration with redox-active molecules^3^. MOFs’ high internal surface areas are advantageous for gas sorption^5^ or chemical sensors^6^, as demonstrated with H_2_^7^, CO_2_^8^, N_2_ and methane^9^. MOFs built with tetracyanochinodimethan (TCNQ) as the linker exhibit an excellent selective uptake of O_2_ and NO^10^ in comparison to N_2_, CO, CO_2_, C_2_H_2_ or Ar, and separation of benzene from cyclohexane^11^, as a result of guest-host charge transfer interactions. Furthermore, nanoscale voids in MOFs can accommodate molecules as large as fullerenes^12, 13^.

One of the challenges towards their electronic applications is to make MOFs optimally conductive to be used as electronic components^14^. Only recently, electrically conductive MOFs were realized through covalent interactions^15^ or doping with TCNQ, a redox active molecule^16^.

**Figure 1:**
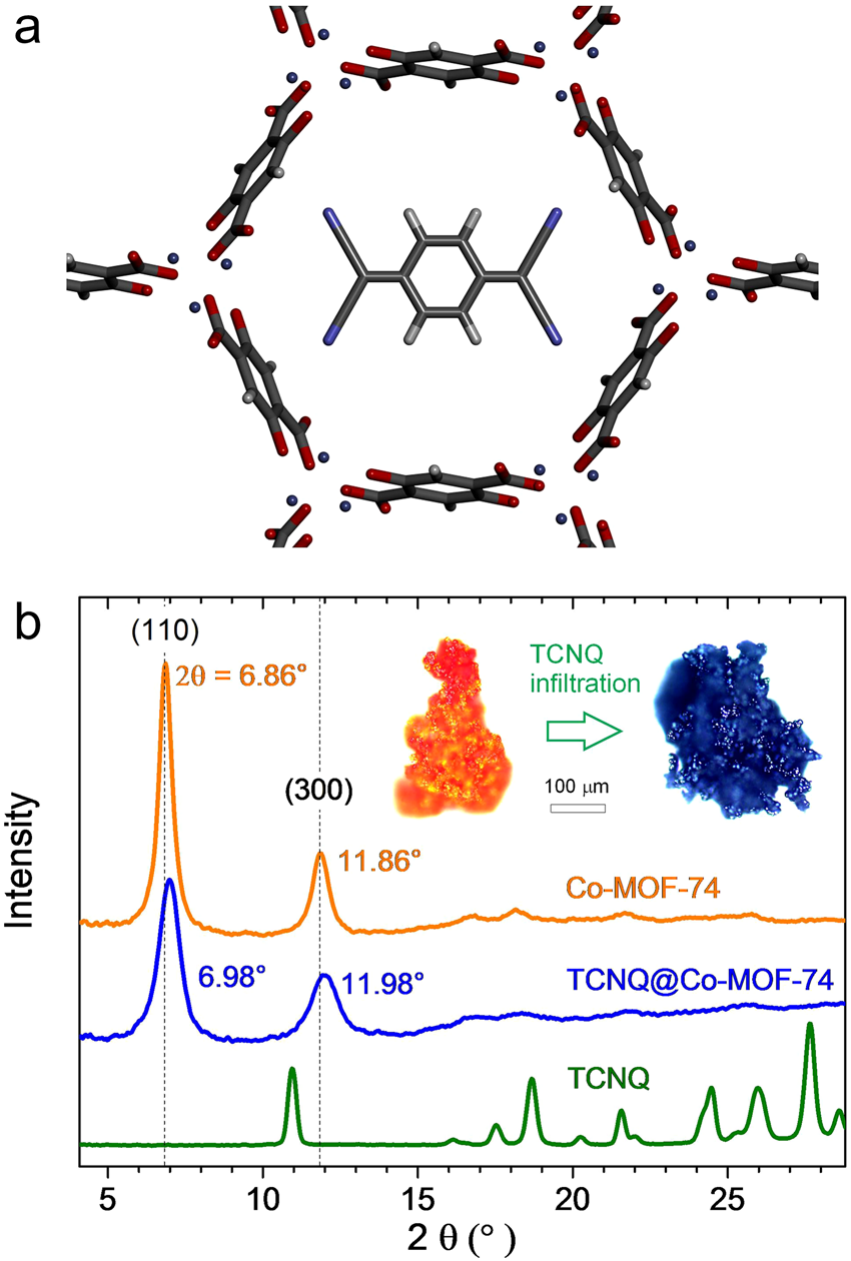
(**a**) A diagram of a Co-MOF-74 framework encapsulating a TCNQ molecule. (**b**) X-ray diffraction profiles of Co-MOF-74 and TCNQ@Co-MOF-74 microcrystallites in comparison to TCNQ powder. The dashed vertical lines are the first two diffraction lines expected for Co-MOF-74^18^. Inset: optical micrographs of microcrystallites before (left) and after (right) the infiltration with TCNQ.

In this article, we study Co-MOF-74 (also called Co-CPO-27 or Co2(dobdc)) crystals^17^ in various forms from nanocrystals to microcrystals and thin films. Infiltrated with TCNQ (see the diagram in Fig. 1a), charge transfer between the host framework and the guest molecule leads to advanced optical and electrical transport properties. Based on the enhanced electronic conduction of the charge-transfer MOF as well as on its extended optical absorption profile covering the entire visible range, we demonstrate their resistive photo- and gas-sensing capabilities, both of which are key properties for future electronic applications of MOFs.

## Results and Discussion

We synthesize Co-MOF-74 in different forms as nanocrystalline, microcrystalline assemblages and as thin films, which we show to be successfully doped by TCNQ. This allows thorough characterization and complementary analysis of the physical properties as well as TCNQ-doped Co-MOF-74 (TCNQ@Co-MOF-74) films to be tested as resistive sensors.

### Structure

X-ray diffraction profiles for Co-MOF-74 microcrystals before and after infiltration with TCNQ are plotted with respect to 2Θ in Fig. 1b. The peaks at 6.86° and 11.86° indexed to (1 1 0) and (3 0 0) with ${{\rm{\Theta }}}_{\mathrm{(300))}}{/{\rm{\Theta }}}_{\mathrm{(110)}}\sim \sqrt{3}$ originate from the hexagonal structure. The dashed vertical lines are the first two diffraction lines expected for Co-MOF-74^18^. The corresponding lattice spacings are 0.645 and 0.375 nm, respectively. With the inclusion of TCNQ, the peaks get slightly upshifted and broader, indicating a contracted and distorted lattice structure, respectively (see SI, section 1). The average crystal size estimated from the width of the (1 1 0) diffraction peak using the Scherrer equation is about 20 nm for the Co-MOF-74 and 11 nm for the TCNQ@Co-MOF-74.

**Figure 2:**
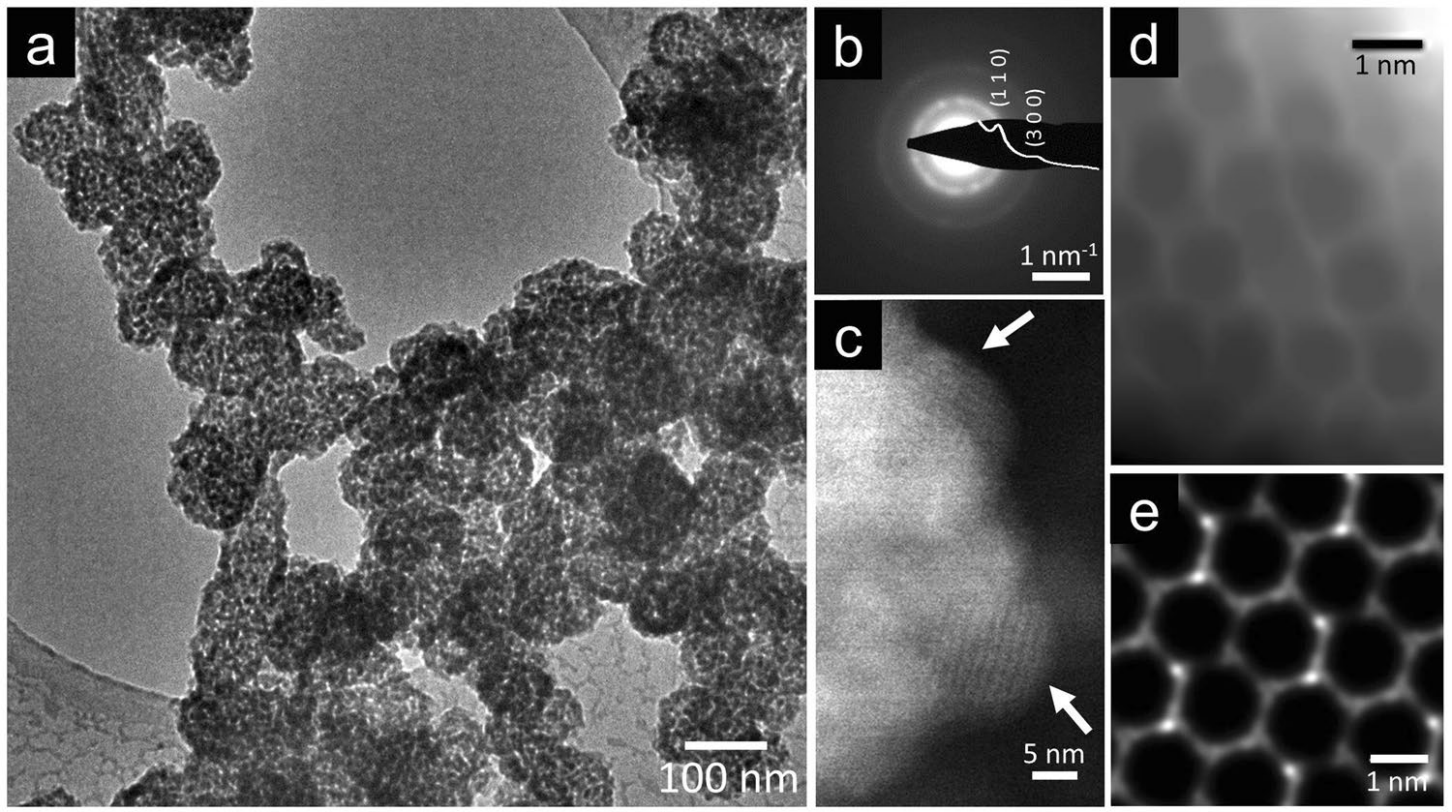
(**a**) BF TEM image of agglomerated Co-MOF-74 nanocrystals. (**b**) SAED pattern of such nanocrystals in which the extracted radial intensity profile in the overlay confirms their Co-MOF-74 structure^18^. (**c**) ADF STEM image of a nanocrystal agglomerate such as in (**a**) at higher magnification. The observed lattice fringes within the individual nanocrystals (marked by arrows) are consistent with Co-MOF-74. (**d**) ADF STEM image (Gaussian blurred and minimum filtered) consistent with the pore structure of a Co-MOF-74 nanocrystal as in (**c**)﻿. (**e**)﻿ STEM image simulation of a Co-MOF-74 crystal viewed along the [001] zone axis (rotated by −18° in plane to match (**d**). Further details on TEM/STEM results are given in SI, section 2.

A bright field (BF) transmission electron microscopy (TEM) image of nanocrystalline Co-MOF-74 in Fig. 2a shows the assemblage of individual nanoparticles into larger agglomerates (50–180 nm). The selected area electron diffraction (SAED) in Fig. 2b confirms the crystallite Co-MOF-74 structure, consistent with the XRD profile in Fig. 1b. Annular dark field (ADF) scanning transmission electron microscopy (STEM) at higher magnification in Fig. 2c further corroborates the crystalline nature of individual nanocrystals of about 4 to 15 nm size. Finally, Fig. 2d visualizes the honeycomb pore structure in one of these Co-MOF-74 nanocrystals, consistent with a Co-MOF-74 crystal viewed along the [001] zone axis, as shown in the image simulation in Fig. 2e (see SI, section 2). Note that characterization of MOFs by TEM/STEM is notoriously challenging because of the high susceptibility of the organic linkers to electron beam induced degradation^19–21^. Our high-resolution microscopy data are the first such available on Co-MOF-74 nanocrystals to date^19, 20, 22^.

### Optical absorption spectroscopy

**Figure 3:**
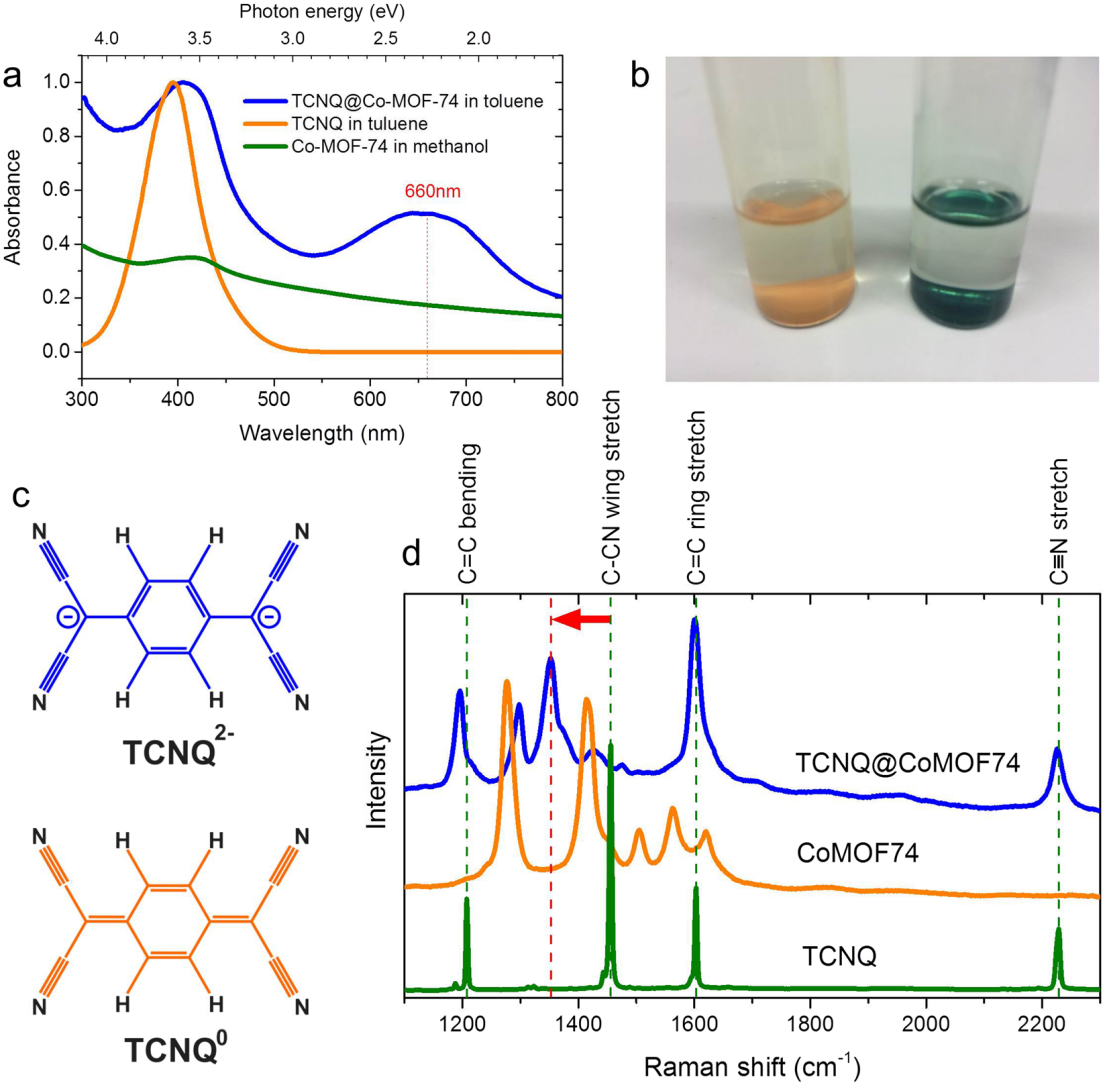
(**a**) Absorption spectra of TCNQ, Co-MOF-74 and TCNQ@Co-MOF-74 nanocrystals. (**b**) A photograph of the glass vials containing Co-MOF-74 (left) and TCNQ@Co-MOF-74 (right) nanocrystals, both in methanol. (**c**) Diagrams for neutral TCNQ^0^ (bottom) and divalent TCNQ^2−^ (top). (**d**) Raman spectra of TCNQ, Co-MOF-74 and TCNQ@Co-MOF-74 microcrystals collected at 633 nm laser wavelength. The dashed vertical lines in green are the frequencies for the four major Raman lines of TCNQ^0^, while that in red is associated to the frequency for red-shifted C-CN wing stretch mode of TCNQ^2−^ in the Co-MOF-74.

Upon infiltration with TCNQ, the MOF’s colour changes from ochre to dark cyan as observed in the optical micrographs in Fig. 3b. As shown in Fig. 3a, neutral TCNQ^0^ in toluene has its giant optical transition peak located at about 395 nm (3.15 eV), associated with TCNQ’s orange colour, while Co-MOF-74 has a weak absorption peak at ~410 nm (3.0 eV). As TCNQ infiltrates Co-MOF-74, a strong new absorption peak emerges at ~660 nm (1.9 eV) in addition to the Co-MOF-74 peak. Accordingly, the optical gap estimated from the onset of the lowest energy peak is reduced by more than 1.0 eV.

The strongest absorption structures of the TCNQ^−^ monomer at around 800 nm^23–26^ are not visible, excluding the presence of monovalent TCNQ. Absorption at ~2 eV (~640 nm) was previously observed as a minor contribution to the absorption profiles of TCNQ^−^ dimers in aqueous solution^23–26^, blue-shifted as compared to the corresponding absorption in TCNQ^−^ monomers. Absorption structures at a similar energy region were also observed in TCNQ^−^ salts in the solid state^26^, but again only as a minor effect associated with locally excited transitions polarized perpendicular to the molecular chain axis^24^.

It is known that the disproportionation occurs in a way that a TCNQ^−^ dimer transforms to a pair of TCNQ^0^ and TCNQ^2−^ ^27^. The divalent TCNQ^2−^ anions in solutions exhibit a large and broad absorption peak at around 2 eV (~640 nm)^27^, along with high energy absorption peaks at about 300 nm^28, 29^. Hence, the 2 eV (~640 nm) peak of TCNQ^−^ dimers observed previously could be the component of disproportionated TCNQ^2−^. Note that the oxidation product of TCNQ^2−^, dicyano-p-toluoyl cyanide (DCTC^−^), has an absorption peak at about 480 nm as observed in electrochemically doped TCNQ^29, 30^ and charge transfer salts^31^, in line with molecular orbital calculations^32^.

Considering the above, the 660 nm peak of TCNQ@Co-MOF-74 can be attributed to TCNQ^2−^, which means that [TCNQ]^2−^ [Co-MOF-74]^2+^ salt is created.

### Raman spectroscopy

Assigning the 660 nm peak to the TCNQ is supported by the fact that Raman response of the TCNQ acceptor is resonance enhanced (see SI, section 3). Figure 3d compares Raman data collected with a laser wavelength of 633 nm (in resonance with TCNQ^2−^) for Co-MOF-74 before and after infiltration with TCNQ as well as that for TCNQ crystals. The major four lines for TCNQ crystals are located at Raman frequencies of 1207.5, 1455.5, 1602.8 and 2228.5 cm^−1^, assigned to the C=CH bending, C-CN wing stretch, C=C ring stretch and C≡N stretch modes, respectively. In TCNQ@Co-MOF-74, those except for the C-CN wing stretch mode are visible at 1195.5, 1600.8, 2226.0 cm^−1^, but the C=CH bending mode frequency is markedly red-shifted by −12 cm^−1^. Other weak peaks can be assigned to frequency-shifted modes of the Co-MOF-74 component, except for the peak at 1352 cm^−1^ with an intensity comparable to the other three TCNQ modes’. Hence, this peak can be attributed to the C-CN wing stretch mode shifted by −103.5 cm^−1^. Indeed, the C-CN wing stretch mode is known to be frequency-shifted by doping as electrons are preferentially accommodated in the wings of TCNQ^33^. Judging from the previously reported frequency shifts for ionic TCNQs, the corresponding doping level is estimated to be ~1.5 ± 0.2 e^−^ per TCNQ (see SI, section 4). This non-integer charge transfer lower than 2 e^−^ per TCNQ, estimated from the optical absorption data in the previous section, could be due to guest-host orbital mixing, as reported previously in Cu_3_ (BTC)_2_ (BTC=benzene tricarboxylate), commonly known as HKUST-1^16^. Note that no sign of DCTC^−^ is observed in the Raman data^28, 29, 34^. We also note that our TCNQ@Co-MOF-74 is stable against environmental changes such as repeated evacuation/venting with inert gas and moderate vacuum annealing at temperatures up to 300 °C, as shown in SI, section 5.

### XPS

**Figure 4:**
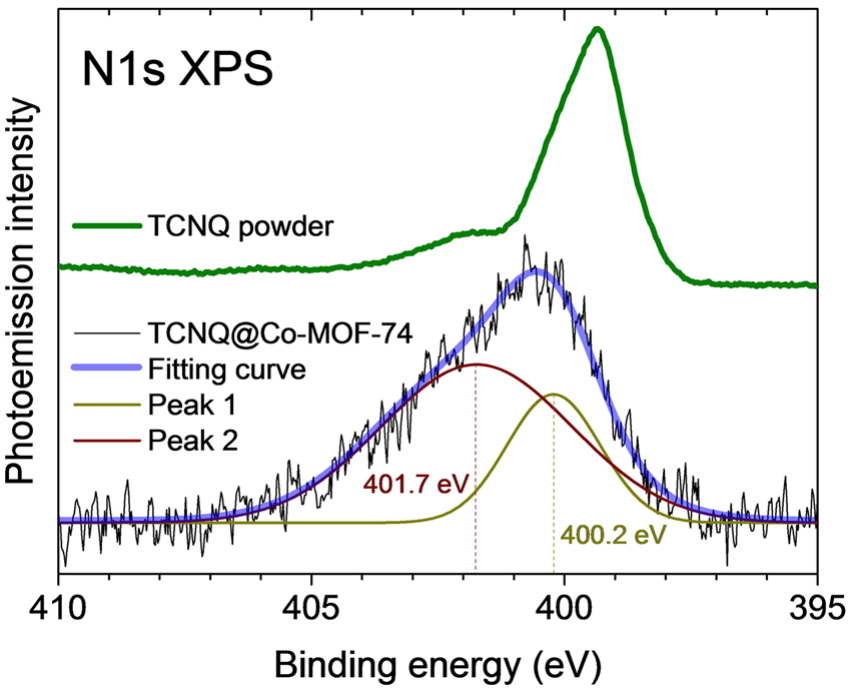
N1 *s* photoemission spectra of TCNQ and TCNQ@Co-MOF-74. The latter is fit with two gaussian peaks.

If there were a guest-host chemical bonding, it could be between oxygen and TCNQ wing as suggested in the copper-based HKUST-1 infiltrating TCNQ^16^. Or, a process like TiO_2_-TCNQ colouration leads to interfacial charge-transfer optical transitions^35^. Figure 4 shows X-ray photoemission spectroscopy (XPS) data at the N1 *s* edge. For neutral TCNQ in powder, the main N1*s* peak is observed at 399.4 eV and a shake-up peak at about 2 eV above the main peak. For the TCNQ in MOFs, the main peak (peak 1) is shifted to 400.2 eV with an enhanced broad shake-up peak (peak 2) at 401.7 eV. This is in contrast to the case of TCNQ^−^/Cu(100)^36^ and lithium-intercalated TCNQ^37^, in which the main peak is shifted towards a lower binding energy and the shake-up feature suppressed. Both observations are attributed to the addition of one electron. An enhanced screening effect due to reduced charge transfer energy Δ_*CT*_ as a result of enhanced electrical conductance through the TCNQ^2−^ should also reduce the binding energy. Hence, molecular orbital changes that possibly involve orbital hybridizations between the nitrogen and the host are likely to explain the energy shifts to higher binding energies. The two N1 *s* splits with area intensities of 0.42 (peak 1) and 0.58 (peak 2) are indicative of two distinguished chemical states of nitrogen due to an asymmetric charge distribution resulting from chemical bonding favouring one side of the TCNQ (see SI, section 6).

### Transport properties

**Figure 5:**
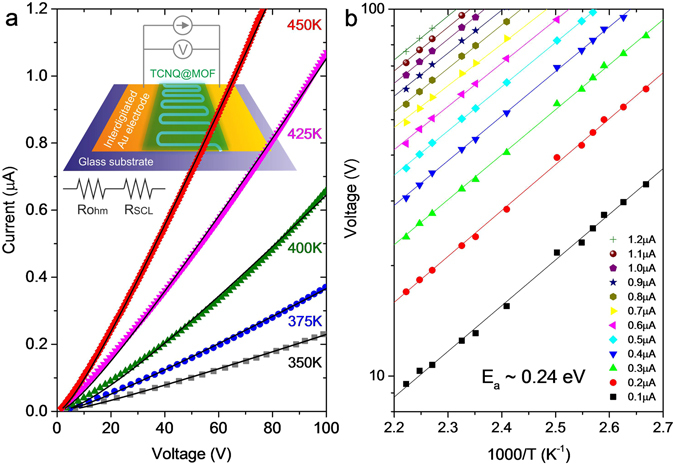
(**a**) Current–voltage (I-V) data for TCNQ@Co-MOF-74 measured at 350, 375, 400, 425 and 450 K, fit to *V* = *αI* + *bI* ^0.5^ (solid curves) assuming that the measured resistance is expressed as ohmic (*I* ∝ *V*_*Ohm*_) and space charge limited ($I\propto {V}_{SCL}^{2}$) resistances connected in series. Inset: a diagram for transport measurements on a TCNQ@Co-MOF-74 film deposited on an interdigitated gold electrode. (**b**) Voltages versus inverse temperature measured at different currents from *I* = 0.1 *μ*A up to 1.2 *μ*A. The solid lines are Arrhenius functions *V*(*T*) ∝ exp (−*E*_α_/*k*_*B*_ *T*) fitting the data well with an activation energy of *E*_*α*_~0.24 eV.

Provided that the electronic gap is reduced by doping, our charge-transfer TCNQ@Co-MOF-74 should become more conductive. While the undoped MOF is as insulating as the glass substrate at temperatures up to 450 K, the resistance of the doped MOF falls into a measurable range. Figure 5a presents the current–voltage (I-V) characteristics of a TCNQ-MOF film grown directly on interdigitated gold electrodes on a glass plate. The data measured at various temperatures in a range from 350 up to 450 K are nonlinear and follow a power-law dependence *I* ∝ *V* ^1.2^ (see SI, section 7.1). For the electrical conduction through metal electrode–dielectric interfaces, the space charge limited (SCL) conduction model^38^ predicts a power-law scaling *I* ∝ *V* ^2^. Assuming that the measured conduction across the TCNQ-MOF device is expressed as ohmic (*V*_*Ohm*_ = *aI*) and SCL (*V*_*SCL*_ = *bI* ^0.5^) resistances connected in series, i.e., *V* = *aI* + *bI* ^0.5^, we can fit the data fairly well, as demonstrated in Fig. 5a. The ratio of *V*_*Ohm*_ to *V*_*SCL*_, apparently scaled by *I* ^0.5^, decreases as the temperature increases, but stays well above one with currents in the sub-*μ*A range (see SI, section 7.2), meaning that the ohmic conduction is dominant. Yet, our TCNQ-MOF is not as conductive as HKUST-1 infiltrated with TCNQ^16^ for which it was suggested that electrical conduction takes place through the conjugated *π* orbital of TCNQ bridging the dimeric copper subunits in the framework. This indicates that the honeycomb structure of Co-MOF-74 doesn’t allow such a continuous conducting path to be established.

In turn, the voltage versus inverse temperature data, measured with various currents up to 1.2 *μ*A, exhibit a linear slope in an Arrhenius plot in accordance with the function *V*(*T*) ∝ exp(−*E*_*α*_/*k*_*B*_ *T*), where *k*_*B*_ is the Boltzmann constant, with carrier injection activation energy *E*_*α*_ = 0.24 eV, as shown in Fig. 5b.

### Sensing

**Figure 6:**
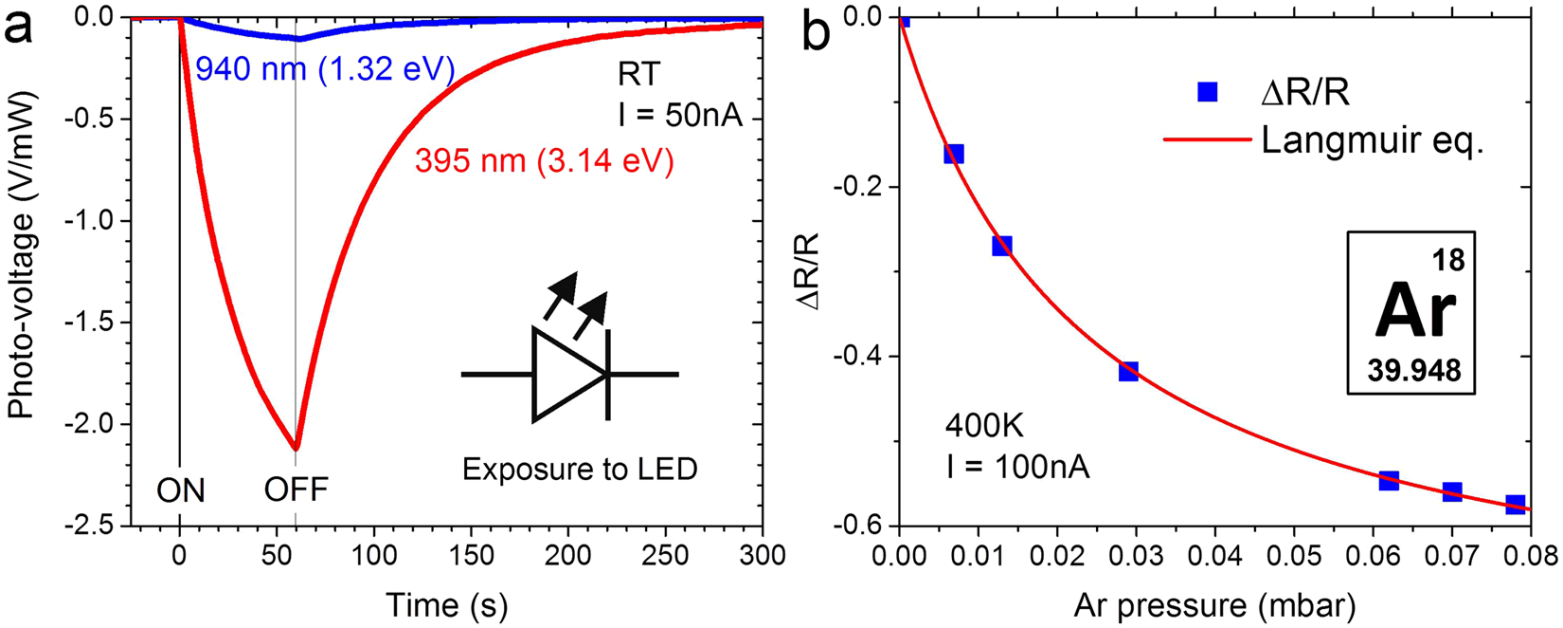
(**a**) Normalized photo-voltage (V/mW) upon UV/IR illumination measured at 50 nA at room temperature. (**b**) Changes in resistance vs argon pressure measured at 100 nA at 400 K.

Our TCNQ–MOF with the enhanced electrical conduction is a good candidate as a resistive sensing element. Figure 6a demonstrates that the resistance at room temperature is markedly reduced upon exposure to UV radiation from a light-emitting diode (LED) with a central wavelength (photon energy) of 395 nm (3.14 eV), while only a subtle reduction (about twenty times smaller) is observed with infra-red LED radiation with a central wavelength (photon energy) of 940 nm (1.32 eV). This is fully consistent with an optical absorption gap of about 1.5 eV.

Furthermore, we have tested our device for gas sorption. Figure 6b shows the resistance of the TCNQ–MOF at 400 K at different pressures of argon. The resistance decreases as argon pressure increases. The response is reversible so that the resistance reverts to the original value when the gas is evacuated. The fact, that the resistance reduction occurs in the same way upon exposures to argon, nitrogen, oxygen and carbon dioxide regardless of their redox properties, means that it is evoked by pressure increase rather than carrier doping. Generally, dissipative heat loss due to an increased number of gaseous molecules in the vacuum chamber leads to enhanced resistances, opposite to what is observed here. The fact that the pressure (*P*) dependence follows Langmuir’s adsorption model for surface coverage, *θ* = *P*/(*P* + *P*_0_), where *P*_0_ = 0.024 mbar, indicates that the device responds to the physical adsorption onto the TCNQ–MOF.

## Conclusion

Charge transfer between molecular components is central to metallic conduction and superconductivity in charge-transfer complexes and doped organic molecules^39–41^. We have shown that the guest-host charge transfer and the orbital hybridization induce the electrical conduction of MOFs. Based on the TCNQ-doped MOF device we have demonstrated ‘resistive’ photo- and gas-sensing. The implementation of doped MOFs as highly porous electrodes would pave the way towards advanced technology that exploits extraordinary physical and chemical properties of MOFs.

## Experimental

### Sample preparation

Co-MOF-74 microcrystals and thin films were synthesized at room temperature as follows. 20 *μ*mol of cobalt (II) acetate tetrahydrate dissolved in 4 mL of methanol was drop cast on top of 10 *μ*mol of 2,5-dihydroxyterephthalic acid (dhtp) in 4 mL of N,N-dimethylformamide (DMF) in a 10 mL glass vial. After a week, microcrystals grown on the glass wall at the interfacial level were collected, washed with DMF and then with methanol. For transport measurements integrated gold electrodes were deposited on a glass plate that was then placed at the boundary between the above two solutions so that a thin film was grown directly on the electrode in a day.

Finer crystals were synthesized by mixing 20 *μ*mol of cobalt (II) acetate tetrahydrate in 4 mL of methanol and 10 *μ* mol of 2,5-dihydroxyterephthalic acid (dhtp) in 4 mL of methanol. Nanocrystals (ochre colour) that instantly precipitated and fell on the bottom of the vial were filtered out and rinsed with methanol for further processes.

The crystal color changes immediately from ochre to dark green as Co-MOF-74 materials were immersed in a saturated toluene solution of tetracyanoquinodimethane (TCNQ: chemical formula C_12_H_4_N_4_). The solvent was exchanged several times before the sample were (filtered out and) washed with toluene after one or several days of immersion. Crystals were kept in vacuum for at least half an hour before Raman and XRD measurements. Absorption measurements were done on nanocrystals dispersed in toluene.

### X-ray diffraction

XRD measurements were carried out with crystals stuck on a transparent adhesive tape or encapsulated in a thin aluminium foil placed in vacuum at room temperature using a Bruker Nanostar equipped with a pinhole camera, a 2D gas detector (Vantec 2000) and an image plate (Fuji FLA 7000) for simultaneous measurement of small-angle and wide-angle X-ray scattering patterns. The X-ray patterns were radially averaged to obtain the scattering intensities in dependence on the scattering vector 4*π* sin(*θ*)/*λ*, where 2*θ* is the scattering angle and *λ* = 0.1542 nm the X-ray wavelength. The XRD profiles of the adhesive tape and aluminium were measured and subtracted from the data.

### Transmission electron microscopy

For transmission electron microscopy (TEM) and scanning transmission electron microscopy (STEM) as-synthesized MOF material was deposited onto amorphous carbon TEM grids with regular hole arrays (Quantifoil) by drop casting or (for finer dispersion) spraying from solution. TEM employed a Philips CM200 at 200 kV electron acceleration voltage, acquiring bright field (BF) TEM and selected area electron diffraction (SAED) data. STEM was done in an aberration corrected Nion UltraSTEM100 at electron acceleration voltages of 60 kV and 100 kV. For STEM an annular dark field (ADF) detector (40–200 mrad) was used. STEM images were simulated using QSTEM software^42^. Analysis of SAED patterns and QSTEM simulation used the Inorganic Crystal Structure Database (ICSD) entry 270293 for Co-MOF-74^18^.

### Raman spectroscopy

Raman spectroscopy measurements were performed at room temperature using a Horiba Jobin Yvon LabRAM spectrometer equipped with a HeNe laser that operates at a wavelength of 633 nm (*E*_*ex*_ = 1.96 eV) and Coherent Innova 70 ion laser at a wavelength of 458, 488, 514.5, 531, 568, 647 nm (*E*_*ex*_ = 2.71, 2.54, 2.41, 2.34, 2.18, 1.92 eV).

### X-ray photoemission spectroscopy

X-ray photoemission spectroscopy experiments were carried out at room temperature using a laboratory setup equipped with a Scienta RS4000 hemispherical analyser and an monochromatic Al K_α_ source (1486.6 eV). Nanocrystals of non-doped or doped MOFs were drop cast from a methanol or toluene solution, respectively, onto a gold substrate.

### Electrical transport measurements

Arrays of five interdigitated gold electrodes were deposited in vacuum onto a glass plate using a shadow mask (Ossila Limited, Interdigitated 18 mm × 50 *μ*m) and connected in parallel with one another. Thin films of non-doped or doped MOFs synthesized directly onto the electrodes were mounted in a vacuum chamber equipped with a heater, a quartz optical window and a gas inlet. Before transport measurements, samples were degassed in vacuum at 450 K until no change in resistance was observed. Keithley Model 6514 Electrometer and Model 6221 AC and DC current source were used for voltage measurements with the temperature controlled by Lakeshore Model 330. A UV light-emitting diode (LED) lamp with a central wavelength (photon energy) of 395 nm (3.14 eV) and a infra-red LED lamp with a central wavelength (photon energy) of 940 nm (1.32 eV) were used for photo-resistive sensing experiments.

## Electronic supplementary material

Supplementary information
