# Supplementary material for: Doping of metal–organic frameworks towards resistive sensing
Source: Sci Rep. 2017 May 26;7:2439. doi: 10.1038/s41598-017-02618-y (PMC5446391; doi:10.1038/s41598-017-02618-y)
Supplement: Supplementary file 1 — Supplementary information [file 41598_2017_2618_MOESM1_ESM.pdf]

Supplementary information (SI)

Doping of metal–organic frameworks towards resistive sensing

Hidetsugu Shiozawa,[1*] Bernhard C. Bayer,[1] Herwig Peterlik,[1] Jannik C. Meyer,[1] Wolfgang Lang,[1] Thomas Pichler[1]

[1]Faculty of Physics, University of Vienna, Boltzmanngasse 5, 1090 Vienna, Austria

*To whom correspondence should be addressed; E-mail: hidetsugu.shiozawa@univie.ac.at

1 The effect of heating as observed with X-ray diffraction

In-situ X-ray diffraction measurements were done upon annealing the TCNQ@Co-MOF-74 at temperatures up to 300 °C, Fig. S1. The MOF's (1 1 0) and (3 0 0) peaks are shifted to higher angles upon annealing at 200 °C above which shifts are subtle up to 300 °C. The post-annealing measurement at 30 °C confirms that the shifts are irreversible. This lattice contraction that is not observed with the empty Co-MOF-74 can be attributed to the guest-host electrostatic attraction emerging due to the removal of toluene.

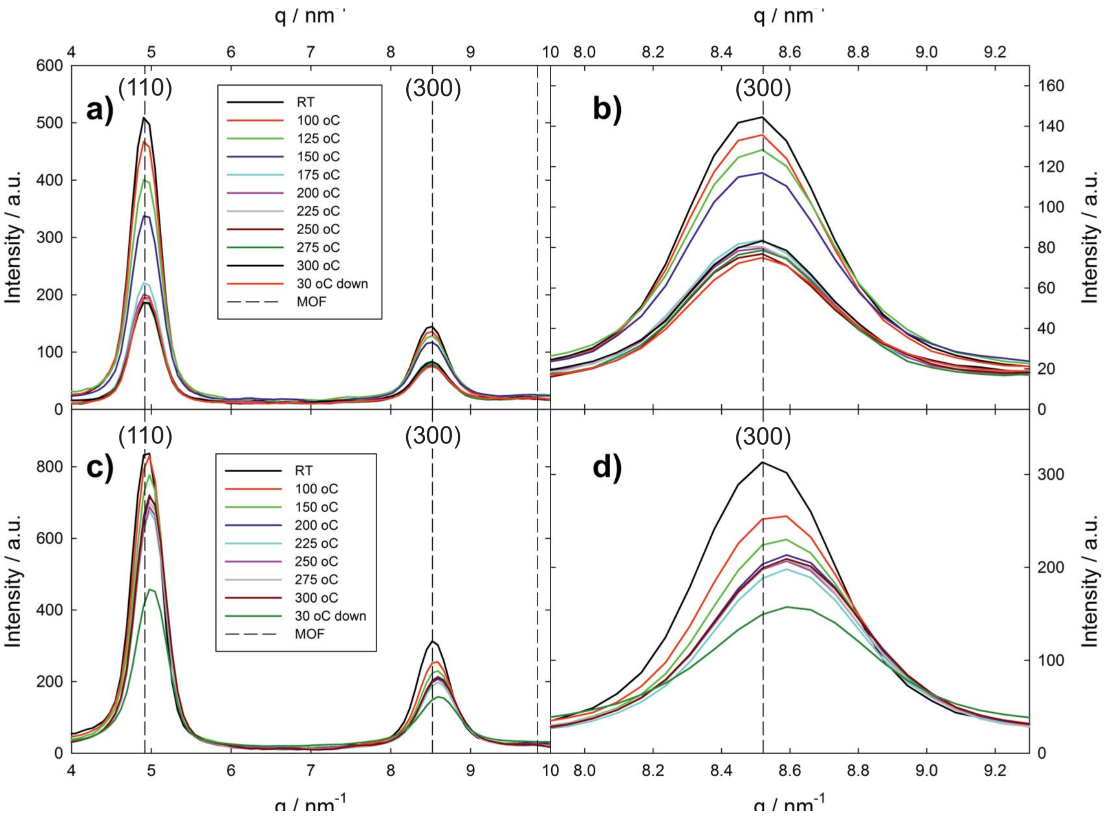

Figure S1: X-ray diffraction profiles from in-situ measurements in vacuum and heated to 300 °C. a) Co-MOF-74, b) Enlarged (3 0 0)-peak of Co-MOF-74 to show that there is no temperature dependence, c) TCNQ@Co-MOF-74, d) Enlarged (3 0 0)-peak of TCNQ@Co-MOF-74 clearly shows a peak shift.

2 Transmission electron microscopy analysis

Figure S2(a) Bright field (BF) transmission electron microscopy (TEM) image of agglomerated Co-MOF-74 nanocrystals, at a higher magnification than in Fig. 2a. (b) Annular dark field (ADF) scanning transmission electron microscopy (STEM) image of a nanocrystal agglomerate (same region as in Fig. 2c, but at larger field of view). The observed lattice fringes within the individual nanocrystals are consistent with Co-MOF-74 [1]. (c) Selected area electron diffraction (SAED) pattern (same as in Fig. 2b) of Co-MOF-74 nanocrystals in as-deposited state, i.e., electron beam exposure < 1s. (d) Radial SAED profile extracted from (c) which is indexed to Co-MOF-74 [1]. (e) SAED pattern from the same location as (c) after 5 min. electron beam exposure, showing complete electron beam induced degradation of the MOF structure [2, 3]. (f) Unprocessed (left), Gaussian blurred (middle) and Gaussian blurred and minimum filtered (right) ADF STEM data corresponding to Fig. 2d at a larger field of view. Note that the data is slightly astigmatic, but largely matching a Co-MOF-74 crystal viewed along the [001] zone axis [1]. The pore structure had disappeared completely in the subsequent STEM scan frame (< 1s later), which is already indicated by beginning pore collapse in the center of the image. This again highlights the high susceptibility of MOF structures to electron beam induced degradation [2, 3]. (g) QSTEM image simulation[4] of a Co-MOF-74 crystal [1] (thickness 2 unit cells) viewed along the [001] zone axis (rotated by -18° in plane to match the orientation of Fig. 2d), as simulated (left) and after an additional Gaussian blur (right, as in Fig. 2e) to account for STEM imaging imperfections. Analysis of SAED patterns and QSTEM simulation used the Inorganic Crystal Structure Database (ICSD) entry 270293 for Co-MOF-74 [1].

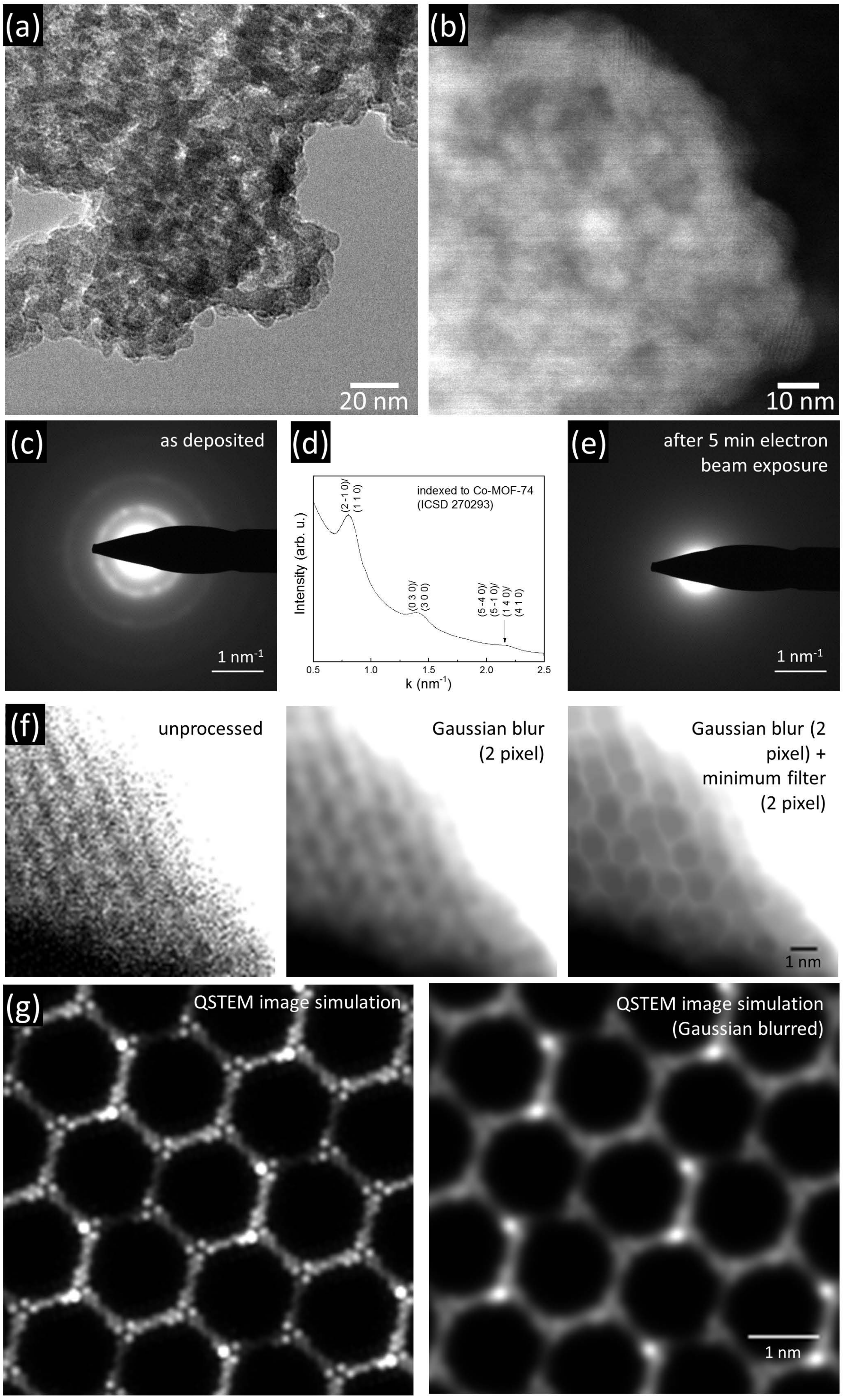

(a)
20 nm
(b)
10 nm
(c)
as deposited
1 nm-1
(d)
indexed to Co-MOF-74
(ICSD 270293)
(2 -1 0)/
(1 1 0)
(0 3 0)/
(3 0 0)
(5 -4 0)/
(5 -1 0)/
(1 4 0)/
(4 1 0)
Intensity (arb. u.)
k (nm-1)
(e)
after 5 min electron
beam exposure
1 nm-1
(f)
unprocessed
Gaussian blur
(2 pixel)
Gaussian blur (2
pixel) +
minimum filter
(2 pixel)
1 nm
(g)
QSTEM image simulation
QSTEM image simulation
(Gaussian blurred)
1 nm

Figure S2:

3 Resonant Raman spectroscopy

Raman spectroscopy data for TCNQ@Co-MOF-74 collected at laser wavelengths of 458, 488, 514.5, 531, 568, 633 and 647 nm exhibit strong resonance effects, as revealed in Fig. S3. At 458 nm that falls within the absorption peak at around 410 nm, the MOF's Raman lines are visible as major components, while as the wavelength reaches the absorption peak at around 660 nm, the TCNQ lines are dominant. This means that the 410 nm and 660 nm absorption peaks are of the Co-MOF-74 host and TCNQ guest, respectively. In the manuscript, the charge state of the TCNQ is discussed with the Raman data taken at 633 nm.

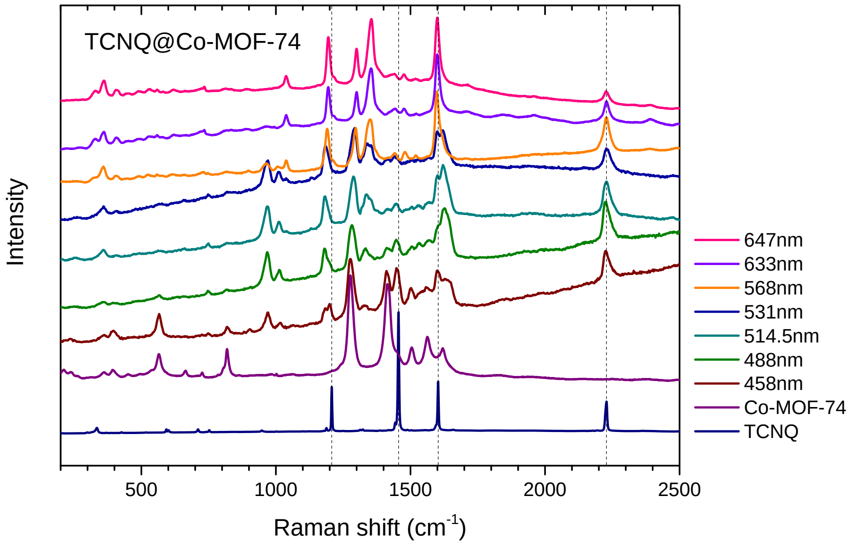

Figure S3: Raman spectra of TCNQ@Co-MOF-74 collected at laser wavelengths of 458, 488, 514.5, 531, 568, 633 and 647 nm, with those of Co-MOF-74 and neutral TCNQ measured at 633 nm.

4 C-CN wing stretch mode of ionic TCNQs

In Fig. S4, Raman frequencies of the predominant C-CN wing stretch mode reported in four previous studies are plotted against the ionic state of TCNQ, together with fitting lines. Dashed horizontal lines at 1455.5 and 1352 cm^{-1} are the C-CN wing stretch mode frequencies for the neutral TCNQ and the ionic TCNQ encapsulated in the Co-MOF-74 measured in the present study. Judging from the intersects between the 1352 cm^{-1} line and four fitting lines, we evaluate the charge state of the TCNQ in the Co-MOF-74 in a range of 1.5 ± 0.2 e^- per TCNQ.

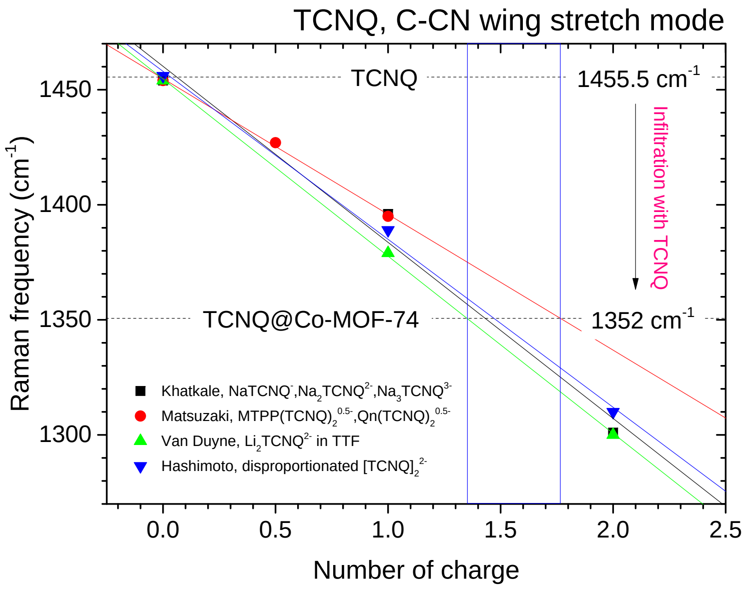

Figure S4: Raman frequencies of the predominant C-CN wing stretch mode plotted against the ionic state of TCNQ, reproduced from Refs.[5, 6, 7, 8].

5 The effect of heating as observed with Raman spectroscopy

Figure S5 shows Raman spectra collected at 633 nm before and after XRD measurements in which the TCNQ@Co-MOF-74 sample was heated in vacuum at temperatures up to 300 °C. All major Raman lines assigned to those of $TCNQ^{2-}$ are visible after heating at 300 °C, proving that TCNQ molecules stay inside the Co-MOF-74.

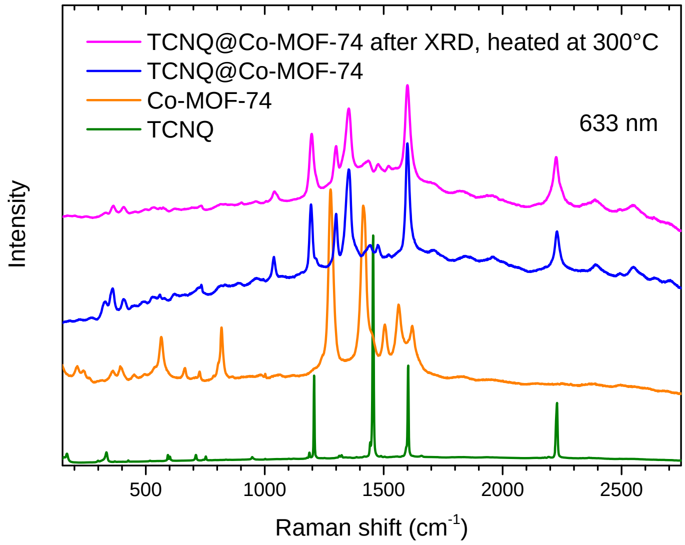

Figure S5: Raman spectra of TCNQ@Co-MOF-74 before and after heating at 300 °C in vacuum, with those of Co-MOF-74 and neutral TCNQ. All data were collected at 633 nm.

6 X-ray photoemission spectroscopy at the Co2p edge

In the Co-MOF-74, cobalt ions in a square pyramidal coordination of oxygen atoms are exposed to the hexagonal voids that accommodate TCNQ. As in other metal-TCNQ salts, TCNQ could be stabilized in the MOF by electron transfer from the cobalt ions or the linker. In the following, we study electronic states of cobalt and nitrogen with regard to the intermolecular charge transfer by means of X-ray photoemission spectroscopy (XPS).

First, taking the photoemission cross section into account, we estimate atomic concentration of nitrogen relative to cobalt, $n_{N/Co}$ ~0.8. This corresponds to a number concentration of TCNQ to cobalt of ~0.2 as one TCNQ has four nitrogen atoms. Considering that the theoretical number ratio of linker molecule to cobalt in the Co-MOF-74 is 0.5, the MOF structure contains a large number of TCNQ, about 40% of the linker in mole.

XPS data at the Co $2p$ edge is plotted in Fig. S6. For the Co-MOF-74, the spectral shape and binding energy are similar to those typically observed for some well-studied Co(II) and Co(II,III) oxides [9, 10] and Co(III) oxides [11, 12]. Upon infiltration with TCNQ, the edge is shifted to a higher binding energy. The spectral shape and binding energy are similar to those of cobalt(II) fluoride [13]. The peak shift didn't change with varying the X-ray intensity, justifying that it is not caused by charging.

The electron configuration of neutral Co^0, divalent Co^{2+} and trivalent Co^{3+} ions are $3d^7 4s^2$, $3d^7 4s^0$ and $3d^6 4s^0$, respectively. Considering the ligand state L, the initial state of the $2p$ photoemission in $3d$ transition metals can be expressed as a linear combination of $3d^n$, $3d^{n+1}L^{-1}$ and $3d^{n+2}L^{-2}$ states, where $n = 7$ and 6 for Co^{2+} and Co^{3+}, respectively. When the orbital hybridization effect and crystal fields are omitted, the energies of the two excited states $3d^{n+1}L^{-1}$ and $3d^{n+2}L^{-2}$ are Δ_{CT} and $\Delta_{CT} + U_{dd}$ above the energy of the ground state $3d^n$, where Δ_{CT} is the charge transfer energy between the $3d$ and the ligand state L, and U_{dd} the Coulomb energy between $3d$ electrons.

A peak fit analysis shows two or three spin-orbit coupled pairs with $2p_{5/2}$ ($2p_{3/2}$) peaks located at 783.62 and 787.83 eV (799.12 and 803.32 eV) with a spin-orbit splitting energy of Δ_{LS} = 15.5 eV with TCNQ and 781.34, 783.64 and 786.55 eV (797.41, 800.26 and 803.53 eV) without TCNQ. The corresponding final state is a linear combination of $c^{-1}3d^n$, $c^{-1}3d^{n+1}L^{-1}$ and $c^{-1}3d^{n+2}L^{-2}$ states with energies of the second (third) state lowered by U_{cd} ($2U_{cd}$) from the respective initial state energies. Apparently, the relative binding energies depend on the degree of screening of the core hole by charge transfer, i.e., Δ_{CT}, relative to the coulomb potential, U_{cd}. It was reported that $\Delta_{CT} < U_{cd}$ in CoF_2 while $\Delta_{CT} > U_{cd}$ in some cobalt alloys with Co^{2+} (n = 7) such as $CoCl_2$ and $CoBr_2$. In the former case, the final state of the lowest energy is the

$c^{-1}3d^7$ state that lowers the Co $2p$ binding energies, as observed in CoF_2, while in the latter case, it is one of the other states.

In the case of Co-MOF-74 in which cobalt ions are reportedly Co^{2+} $3d^7$ high spin state [1], the final state is a linear combination of $c^{-1}3d^7$, $c^{-1}3d^8L^{-1}$ and $c^{-1}3d^9L^{-2}$. Tentative assignments of the spin-orbit splits in the case of Co^{2+} $3d^7$ with $\Delta_{CT} < U_{cd}$ are given in Fig. S6. The number of spin-orbit splits is reduced from three to two by infiltration with TCNQ, possibly due to changes in Δ_{CT}, ligand field and/or valence number.

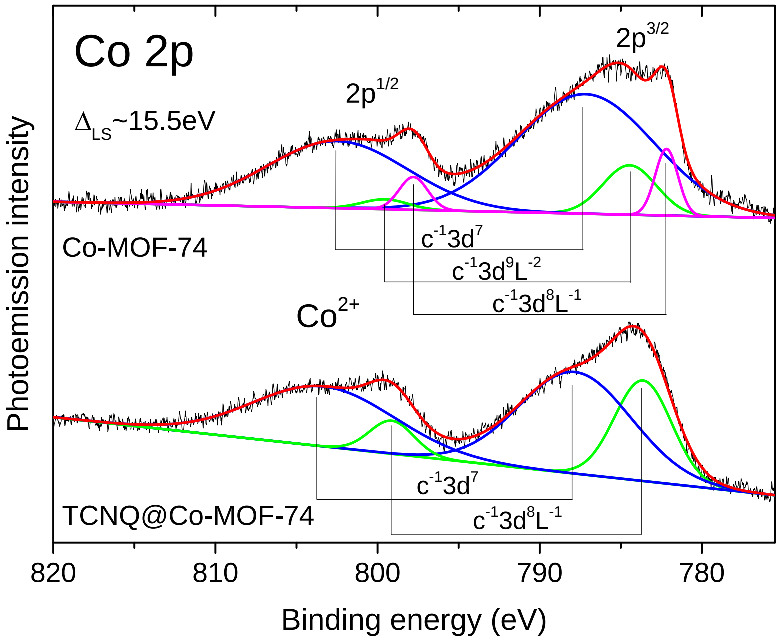

Figure S6: Co 2p photoemission spectra of Co-MOF-74 and Co-MOF-74 infiltrated with TCNQ (TCNQ@Co-MOF-74).

7 Current–voltage characteristics

7.1 I–V curves in the double logarithmic scale

In Fig. S7, the current–voltage (I-V) characteristics of a TCNQ-MOF film are plotted in the double logarithmic scale. The data measured at 350, 375, 400, 425 and 450 K follow a power-law dependence $I \propto V^{1.2}$.

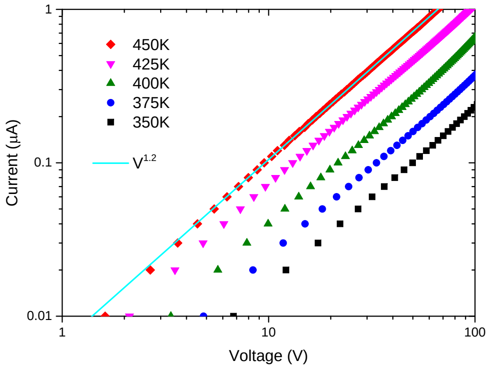

Figure S7:

7.2 Ohmic vs space charge limited conduction

As shown in Fig. S8, the ratio of $V_{Ohm} = aI$ to $V_{SCL} = bI^{0.5}$ is $V_{Ohm}/V_{SCL} = a/b \times I^{0.5}$, and V_{Ohm} is much larger than V_{SCL} over the whole range of current measured.

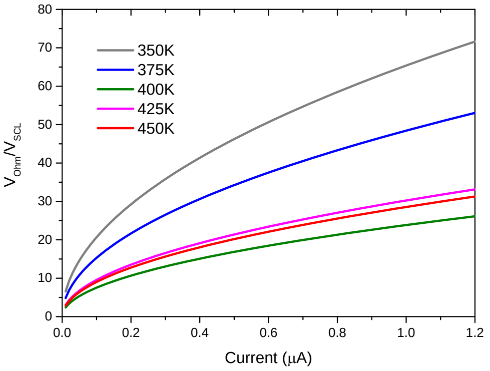

Figure S8:

References

[1] Dietzel, P. D. C., Morita, Y., Blom, R. & Fjellvag, H. An in situ high-temperature single-crystal investigation of a dehydrated metal-organic framework compound and field-induced magnetization of one-dimensional metaloxygen chains. *Angewandte Chemie-international Edition* **44**, 6354–6358 (2005).

[2] Diaz-Garcia, M., Mayoral, A., Diaz, I. & Sanchez-Sanchez, M. Nanoscaled m-mof-74 materials prepared at room temperature. *Crystal Growth & Design* **14**, 2479–2487 (2014).

[3] Mayoral, A., Sanchez-Sanchez, M., Alfayate, A., Perez-Pariente, J. & Diaz, I. Atomic observations of microporous materials highly unstable under the electron beam: The cases of ti-doped alpo4-5 and zn-mof-74. *Chemcatchem* **7**, 3719–3724 (2015).

[4] Koch, C. T. *Determination of core structure periodicity and point defect density along dislocations.* Ph.D. thesis, Arizona State University, http://adsabs.harvard.edu/abs/2002PhDT........50K (2002). Provided by the SAO/NASA Astrophysics Data System.

[5] Van Duyne, R. P. *et al.* Resonance raman spectroelectrochemistry. 6. ultraviolet laser excitation of the tetracyanoquinodimethane dianion. *Journal of the American Chemical Society* **101**, 2832–2837 (1979).

[6] Khatkale, M. S. & Devlin, J. P. Vibrational and electronic-spectra of the monoanion, dianion, and trianion salts of tcnq. *Journal of Chemical Physics* **70**, 1851–1859 (1979).

[7] Matsuzaki, S., Kuwata, R. & Toyoda, K. Raman spectra of conducting tcnq salts; estimation of the degree of charge transfer from vibrational frequencies. *Solid State Communications* **33**, 403–405 (1980).

[8] Hashimoto, S., Yabushita, A., Kobayashi, T. & Iwakura, I. Real-time measurements of ultrafast electronic dynamics in the disproportionation of [tcnq](2-)(2) using a visible sub-10 fs pulse laser. *Chemical Physics Letters* **650**, 47–51 (2016).

[9] Biesinger, M. C. *et al.* Resolving surface chemical states in xps analysis of first row transition metals, oxides and hydroxides: Cr, mn, fe, co and ni. *Applied Surface Science* **257**, 2717–2730 (2011).

[10] van Elp, J. *et al.* Electronic structure of coo, li-doped coo, and licoo$_2$. *Phys. Rev. B* **44**, 6090–6103 (1991).

[11] Tan, B. J., Klabunde, K. J. & Sherwood, P. M. A. Xps studies of solvated metal atom dispersed (smad) catalysts. evidence for layered cobalt-manganese particles on alumina and silica. *Journal of the American Chemical Society* **113**, 855–861 (1991).

[12] Tuan, A. C. *et al.* Epitaxial growth and properties of cobalt-doped zno on alpha-al2o3 single-crystal substrates. *Physical Review B* **70**, 054424 (2004).

[13] Lu, Y. C., Mansour, A. N., Yabuuchi, N. & Shao-Horn, Y. Probing the origin of enhanced stability of "aipo(4)" nanoparticle coated licoo2 during cycling to high voltages: Combined xrd and xps studies. *Chemistry of Materials* **21**, 4408–4424 (2009).
